# Use of a hybrid digital training approach for hormonal IUD providers in Nigeria: results from a mixed method study

**DOI:** 10.1186/s12913-023-10211-5

**Published:** 2023-11-29

**Authors:** Kristen M. Little, Anthony A. Nwala, Eden Demise, Samantha Archie, Ezechukwu I. Nwokoma, Chinedu Onyezobi, Helen Anyasi, Kayode Afolabi, Funmilola M. OlaOlorun, Kate H. Rademacher, Kendal Danna, Lara Lorenzetti, Jennifer Anyanti, Marya Plotkin

**Affiliations:** 1 Population Services International (PSI), Strategy and Insights Department, 1120 19th, Street NW, Suite 600, Washington, DC 20020 USA; 2 Society for Family Health (SFH) Nigeria, RH/FP Division, Abuja, Nigeria; 3 FHI 360, Health Services Research Department, Durham, NC USA; 4 FHI 360, Product Development and Introduction Department, Abuja, Nigeria; 5 Nigeria Federal Ministry of Health, Reproductive Health Division, Abuja, Nigeria; 6 Evidence for Sustainable Human Development Systems in Africa and the Department of Community Medicine, College of Medicine, University of Ibadan, Ibadan, Nigeria; 7 PSI, Sexual and Reproductive Health Department, Washington, DC USA; 8 FHI 360, Behavioral, Epidemiological, and Clinical Sciences Division, Durham, NC USA; 9 FHI 360, Reproductive, Maternal, Newborn, and Child Health Department, Durham, NC USA

**Keywords:** (Hybrid) Digital training, Family planning, Health care provider training, Hormonal IUD, Nigeria

## Abstract

**Background:**

In Nigeria, in-service trainings for new family planning (FP) methods have typically been conducted using a combination of classroom-based learning, skills labs, and supervised practicums. This mixed-methods study evaluated the feasibility, acceptability, provider competency, and costs associated with a hybrid digital and in-person training model for the hormonal intrauterine device (IUD).

**Methods:**

The study was conducted in Enugu, Kano, and Oyo states, Nigeria, and enrolled FP providers previously trained on non-hormonal IUDs. Participants completed a digital didactic training, an in-person model-based practicum with an Objective Structured Clinical Examination (OSCE), followed by supervised provision of service to clients. Provider knowledge gains and clinical competency were assessed and described descriptively. Data on the feasibility, acceptability, and scalability of the approach were gathered from participating providers, clinical supervisors, and key stakeholders. Training costs were captured using an activity-based approach and used to calculate a cost per provider trained. All analyses were descriptive.

**Results:**

Sixty-two providers took the hybrid digital training, of whom 60 (91%) were included in the study (*n* = 36 from public sector, *n* = 15 from private sector, and *n* = 9 both public/private). The average knowledge score increased from 62 to 86% pre- and post-training. Clinical competency was overall very high (mean: 94%), and all providers achieved certification. Providers liked that the digital training could be done at the time/place of their choosing (84%), was self-paced (79%), and reduced risk of COVID-19 exposure (75%). Clinical supervisors and Ministry of Health stakeholders also had positive impressions of the training and its scalability. The hybrid training package cost $316 per provider trained.

**Conclusions:**

We found that a hybrid digital training approach to hormonal IUD service provision in Nigeria was acceptable and feasible. Providers demonstrated increases in knowledge following the training and achieved high levels of clinical competency. Both providers and clinical supervisors felt that the digital training content was of high quality and an acceptable (sometimes preferable) alternative to classroom-based, in-person training. This study provided insights into a hybrid digital training model for a long-acting contraceptive, relevant to scale-up in Nigeria and similar settings.

**Supplementary Information:**

The online version contains supplementary material available at 10.1186/s12913-023-10211-5.

## Background

Globally, over 200 million women in low- and middle-income countries (LMICs) want to avoid or delay a pregnancy but are not using a modern contraceptive method [1]. Expanding contraceptive access and choice can help address this gap [2]. In 2021, the hormonal intrauterine device (IUD), a long-acting reversible contraceptive (LARC), was added for the first time to product catalogs for United Nations Population Fund (UNFPA) and U.S. Agency for International Development (USAID) [3]. Prior to this, the method has not been widely available in the public sector in LMICs [4]. Following early introduction efforts in pilot settings, several countries in sub-Saharan Africa, including Nigeria, are now poised to scale-up the hormonal IUD as part of a full contraceptive method mix [5, 6]. To scale up the method, family planning (FP) providers require training on high-quality counseling, and device insertion and removal techniques, all in the context of supporting client volunteerism and choice [2].

Traditionally, FP clinical trainings in Nigeria begin with a classroom-based, didactic session consisting of lecture-style learning, followed by practice with pelvic models, which is overseen by trained clinical supervisors. Subsequently, providers complete a practicum providing services to clients, with continued support and guidance by clinical supervisors. Trainees are certified to provide FP services after successfully completing training components up to the nationally defined standard. For providers already skilled in LARCs, such as the copper IUD and implants, fostering competence in the hormonal IUD would typically require 1–2 days of classroom instruction and 2–3 days of hands-on clinical practice.

However, the in-person components of clinical trainings tend to be resource intensive, comprising as much as 50% or more of total costs of introduction of new contraceptives such as Subcutaneous Depot Medroxyprogesterone Acetate (DMPA-SC) self-injectable contraceptives [7]. In the case of the hormonal IUD in Nigeria, in-person training costs were noted to be the highest-cost activity in the introduction of the method, representing 92% of total service delivery costs [8]. Digital (or hybrid digital and in-person) training models offer an alternative to in-person learning, with potential benefits of being cost-saving, as well as self-paced and completed at a trainee’s convenience. Recognizing the potential for digital technologies to increase efficiencies and improve health workforce capacity, the High Impact Practices (HIPs) technical advisory group has identified digital health as a HIP enhancement [9].

The Nigerian Federal Ministry of Health (MOH), donors, implementing partners, practitioners and other policy makers in Nigeria have long been interested in exploring digital training approaches, and the Coronavirus Disease 2019 (COVID-19) pandemic further fueled the interest in use of digital technology [10]. There is evidence on the effectiveness of e-learning on improving health care provider knowledge [11, 12] however, limited evidence exists on the effectiveness of e-leaning on provider behavior which can be measured using different methods such as through objective standardized assessment tools or simulations [13]. In the context of scale up of hormonal IUD in Nigeria and the ongoing COVID-19 pandemic, the current study piloted an approach in which the didactic portion of FP clinical training was conducted online via a digital platform. This study assessed the feasibility, acceptability, and scalability of a hybrid online and in-person training course for hormonal IUD insertion and client management for LARC-trained FP providers in Enugu, Kano, and Oyo states in Nigeria. The findings from this study will be useful for those engaged in the introduction or scale-up of new contraceptive methods, or those considering digital or hybrid training options, in Nigeria or similar settings.

## Methods

This descriptive study employed a mixed-methods approach to assess the hybrid hormonal IUD training model piloted among providers and clinical supervisors, and to estimate the direct costs of the training approach. We also examined user experience, and changes in provider knowledge, and competence. Outcome measures included pre- and post-training knowledge and post-training clinical competency in hormonal IUD service provision. We gathered qualitative data on feasibility and acceptability of the training model. Lastly, we conducted an activity-based costing exercise to assess cost per provider trained using the hybrid digital training approach.

### Hybrid digital training approach

**Fig. 1:**
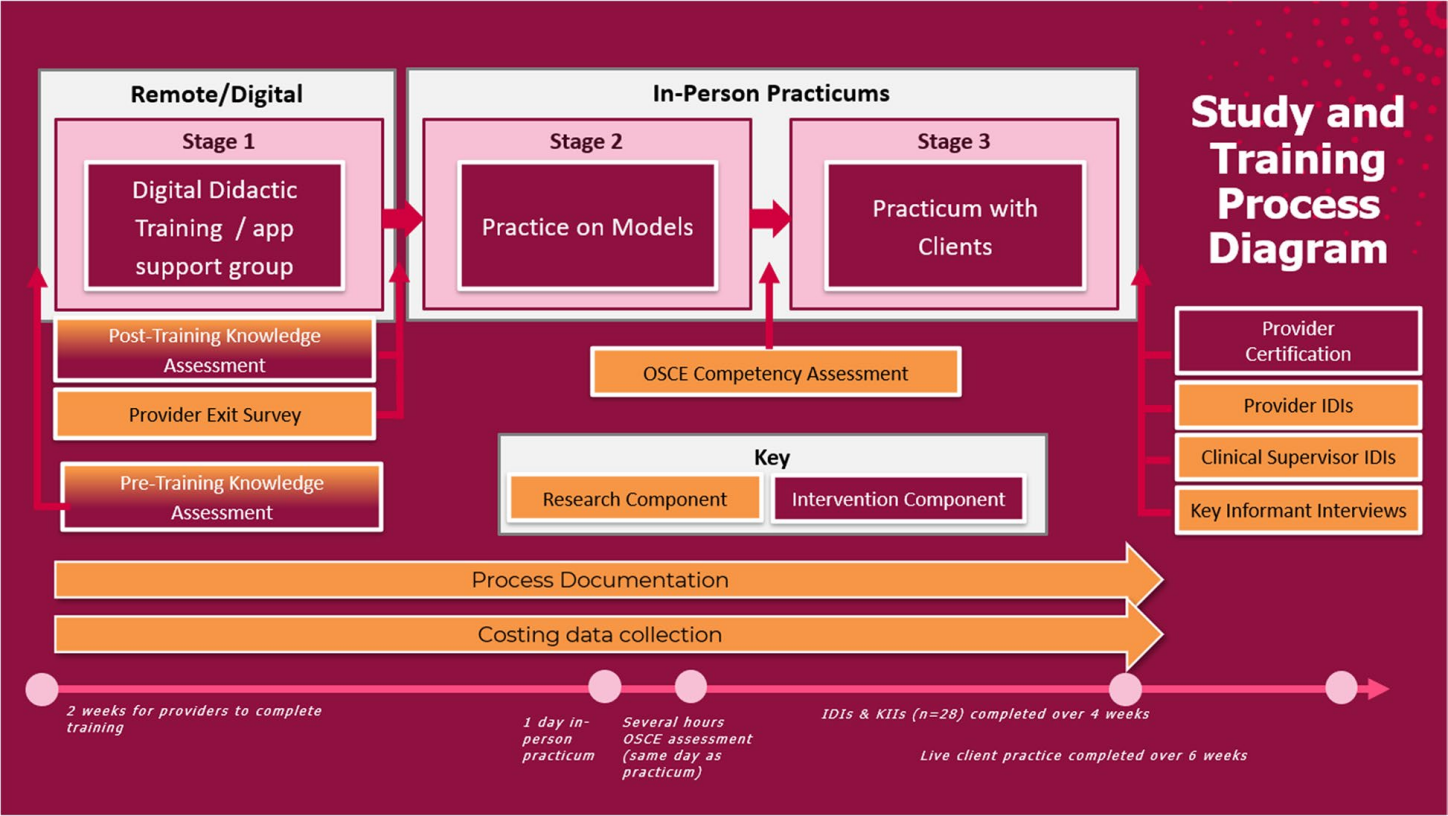
The Hybrid Training Model With Training and Research Components for hormonal IUD providers in Nigeria The training model comprised 3 stages: 1) Digital didactic training over a period of two weeks, inclusive of a WhatsApp-based support group and one virtual question and answer session for all of the trainees; 2) a one-day, in-person intensive practice on models; and 3) provision of the hormonal IUD to a minimum of three clients at trainee’s own health facility setting, supervised by an external clinical supervisor

The hybrid training model comprised 3 stages: 1) Digital didactic training over a period of two weeks, inclusive of a WhatsApp-based support group and one virtual question and answer session for all of the trainees (one session per state); 2) a one-day, in-person intensive practice on models (one per state); and 3) provision of the hormonal IUD to a minimum of three clients at trainee’s own health facility setting, supervised by an external clinical supervisor trained by SFH (Fig. 1). After demonstrating competency providing hormonal IUD to three clients, providers were certified by the Federal MOH as a hormonal IUD provider. More information on the training content and development can be found in the Supplemental Materials. Because providers used their own devices for the training, trainees received an airtime credit worth N5,000 (~ US$11) sent directly to their phones during the digital portion of the training to cover data costs. Trainees were initially given two weeks to complete the digital training modules, and an additional week was provided when several trainees reported that they were not able to complete it within two weeks.

### Study design

The study was conducted between August-October 2021 among FP providers working in private and public sector health facilities in Enugu, Kano, and Oyo states of Nigeria. States were chosen in consultation with the state and Federal MOH based on the presence of SFH franchise facilities; the presence of public sector health facilities able to participate in training; and state MOH’s interest in scaling up hormonal IUD. The purposively selected sample included both public and private sector health care providers (one per participating health facility) previously trained on the copper IUD.^1^ In facilities with > 1 eligible provider, the participating provider was selected by the facility manager based on LARC client volume and interest.

### Study participants

Due to the purposive nature of the participant selection, formal sample size calculations were not performed for this study, and the final sample size was based on budgetary and logistical considerations for the training pilot. We aimed to recruit 30 providers per state, divided equally between the private and public sectors. Providers were eligible to participate in the study if they were a healthcare provider who had delivered FP services in the last year, were previously trained on provision of non-hormonal IUD, had not already received training on the hormonal IUD, and consented to have their anonymized training results shared for study purposes. Providers who did not consent to one or more of the research activities were still able to enroll in the hybrid training, though their data were not shared or analyzed for this research.

A subset of providers was also invited to participate in an in-depth interview (IDI) following the training. For the IDIs, two private sector and two public sector providers were selected per state. Health care providers with low and high post-training knowledge assessment scores were included in each state.

A total of 14 clinical supervisors were involved in the hormonal IUD practicums, though only a subset (*n* = 6) were requested to participate in an IDI. We also conducted key informant interviews (KIIs) with selected stakeholders at the state and national levels. Key informants were eligible for the study if they were a representative of a state or federal health authority, or an implementing partner involved in the design, planning, and implementation of the hybrid training, or who would be involved in potential further scaling of the training.

### Data collection

Study staff attended a one-day training which covered research ethics, including informed consent, familiarity with the study tools and protocols, as well as study logistics. Following completion of digital informed consent, participant trainees completed an online enrollment survey and a pre-training knowledge assessment. The enrollment survey included questions about provider demographics, training history (including previous digital training), and experience providing other long-acting reversible contraceptive methods. Upon completion of the training modules, participant trainees completed a post-training knowledge assessment and exit survey. The exit survey included questions about provider experiences with the training, their perceptions of the training (including training quality and comparison to previous in-person training alternatives), barriers to completing the digital training components, and suggestions for improvement. These assessments were administered through the Kaya e-learning platform. Data from consenting providers were extracted by data managers from the study team from the Kaya database. A study ID was assigned before inclusion into the study datasets so that scores in the training platform could be linked to other study tools. No identifying information was used by the study team in the analysis of the data.

Following the online didactic training, each state held up to two in-person clinical practicum events, which roughly ten of the provider-trainees attended. To assess competency for study purposes, at the end of the one-day clinical practicum, each participant trainee underwent an Objective Structured Clinical Examination (OSCE) in hormonal IUD service provision. In the OSCE assessment, participant trainees were observed conducting counseling for, insertion, and removal of the hormonal IUD using either a standardized patient (counseling) or pelvic models (insertion and removal), while being graded on their performance on the task by observers using a standardized assessment checklist drawn from the national tool. OSCE scores were collected directly onto a tablet by assessors on the day of the assessment and uploaded from the tablets onto a secure server.

Data for the IDIs and KIIs were collected through phone-based interviews which were audio recorded and transcribed. All interviews were conducted in English. Trained interviewers used standardized semi-structured interview guides to conduct the interviews. The quantitative and qualitative study instruments were developed specifically for this study and can be found under Supplementary materials.

### Data analysis

#### Quantitative

Data were analyzed descriptively using Stata v17. Descriptive analyses (means, medians, ranges, and standard deviations) were calculated for pre-/post-training knowledge scores, trainees’ completion of training modules, and OSCE scores, as well as for demographic data (e.g., age, cadre, sector, training, years of experience, etc.) collected through the enrollment survey. Quantitative data from the exit survey, including provider perceptions on the feasibility, acceptability, usability, relevance, and quality of the hybrid training,were also analyzed descriptively.

Knowledge scores were based on an exam administered both before and after the digital training that consisted of 44 multiple-choice and true/false questions. The knowledge score was calculated as a percentage of points received out of the total points possible on the exam. We calculated the mean, median, and range scores for the pre- and post-test, the average difference in scores between the two exams, and the proportion of providers achieving at least a passing score of 80%.

OSCE scores were based on a checklist of 62 items. While each item was worth one point, some of the items on the checklist were required for a provider to pass. OSCE scores were calculated as a percent of the total points possible. We calculated the mean, median, and range of the scores for each OSCE component (counseling, insertion, and removal) and overall, and the proportion of providers achieving at least a passing score of 80% with all required items completed correctly.

#### Qualitative

Transcripts from provider, clinical supervisor, and stakeholder interviews were analyzed with Dedoose v8 qualitative software (SocioCultural Research Consultants, Los Angeles, CA, USA) using pre-defined themes. Themes included feasibility, accessibility, perceived quality, and scalability of the digital training. A codebook was created to define these themes. Two analysts (KL, ED) assigned codes to each transcript, and any differences between analysts was resolved through discussion. After coding, analysis was conducted according to theme, with key quotes extracted and responses tabulated.

#### Costing

We conducted a retrospective activity-based costing exercise to determine the cost per provider trained, intended to capture the financial implementation costs associated with conducting the training. We did not include costs from the perspective of the participant, such as participant time or personal expenditure.

We identified three key areas which characterized costs of the hybrid digital training: 1) involvement in the digital didactic training, 2) involvement in the in-person practicum, and 3) involvement in supervised service provision to clients. For each area, we examined labor, travel, meetings, materials, and other direct costs as distinct cost categories. To calculate labor costs for staff supporting the intervention (including preparing for the training, managing the WhatsApp group, and providing IT support, and facilitating the in-person components), we used a median hourly rate calculated based on information from a SFH human resources salary survey. Travel costs included per diems and travel reimbursements (accommodation, meals, and incidentals). Meeting costs included venue rentals, and materials included IUDs for practice insertion. The Kaya platform subscription cost was included in the materials cost. We disaggregated costs by site by utilizing site-specific costs, where available, or by dividing total costs by state or number of participants, as appropriate. We analyzed these data by generating a total cost per site based on these activities and categories, then cost per provider trained, using the number of participants trained per site.

### Research ethics

This study was reviewed and received an exempt research determination by FHI 360’s Protection of Human Subjects Committee. The study was reviewed and approved by Nigeria’s National Health Research Ethics Committee. Participants provided informed consent for their training data to be used for research purposes, and separate consent to participate in OSCEs and/or IDIs or KIIs.

## Results

### Provider characteristics

**Fig. 2:**
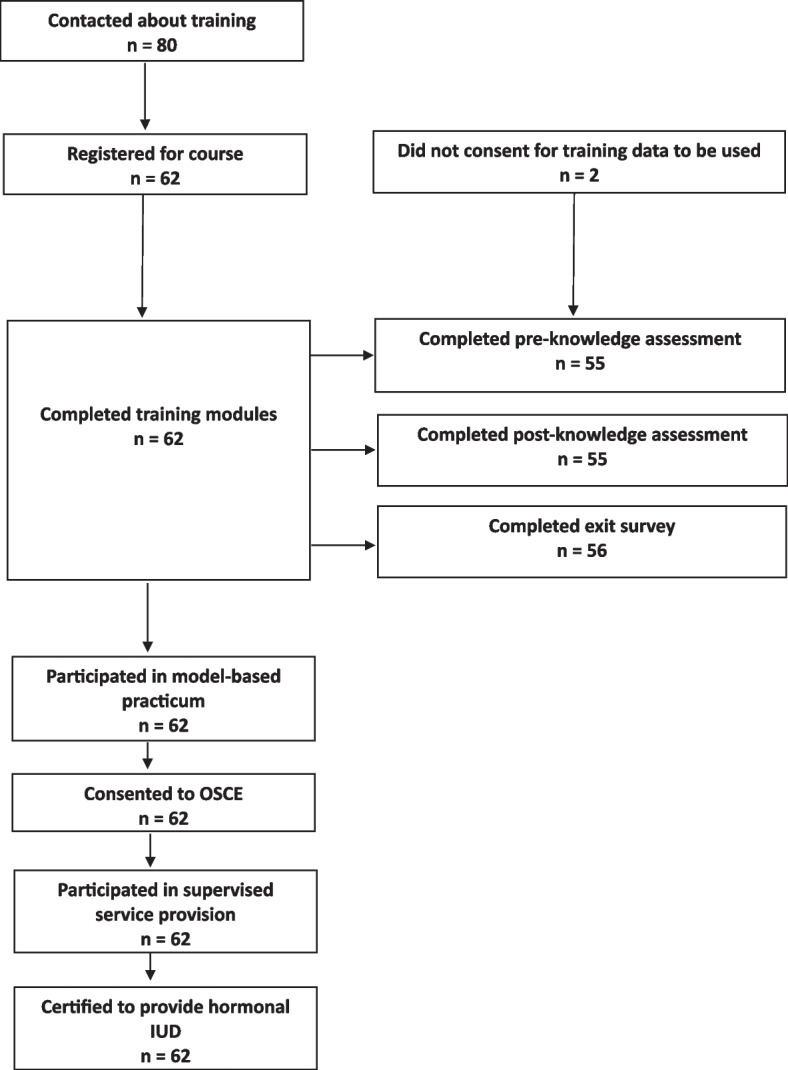
FP providers who participated in different study activity of the hybrid digital training for hormonal IUD in Nigeria Eighty providers were contacted about the training, 62 of whom registered for the course and completed the training modules. Among those who completed the training modules, most completed the pre-knowledge assessment (*n* = 55), post knowledge assessment (*n* = 55) and exit survey (*n* = 56). Also, among those who completed the training modules (*n* = 62), all participated in the model-based practicum, consented to OSCE, participated in supervised service provision, and became certified to provide hormonal IUD

A total of 62 provider trainees completed the digital didactic training within three weeks of enrollment (Fig. 2). Of these, 60 (91%) consented to have their training data used for research purposes, and 56 completed the self-administered exit interview in the Kaya platform. Provider trainees were from Enugu (*n* = 25), Oyo (*n* = 21) and Kano States (*n* = 14) (Table 1). Provider trainees averaged 48 years of age, and most were female (93%). Most trainees were nurses (50%), midwives (33%), or community health officers (11%). Most participants were employed by private sector health facilities (60%) or in both public and private sector health facilities (15%). Providers generally had a significant amount of clinical experience, with most (80%) having been in their current role for 10 years or more. Prior experience with digital training was reported by a quarter (25%) of the study sample.

**Table 1:** Baseline characteristics of sixty family planning providers who were trained on hormonal IUD insertion using a hybrid digital approach in Nigeria

	**Total**	**Enugu**	**Kano**	**Oyo**
	***n* = 60**	***n *= 25**	***n* = 14**	***n* = 21**
	N (%)	N (%)	N (%)	N (%)
**Age in years (range)**	48.2 (21—65)	48.2 (21- 65)	46.5 (30- 57)	49.2 (33- 60)
**Gender**				
Male	4 (6.7)	2 (8.0)	1 (7.1)	1 (4.8)
Female	56 (93.3)	23 (92.0)	13 (92.9)	20 (95.2)
**Cadre**				
Nurse	30 (50.0)	13 (52.0)	3 (21.4)	14 (66.7)
Midwife	20 (33.3)	8 (32.0)	8 (57.1)	4 (19.0)
Community Health Officer	7 (11.7)	3 (12.0)	1 (7.1)	3 (14.3)
Doctor	3 (5.0)	1 (4.0)	2 (14.3)	0 (0.0)
**Sector of employment**				
Public	36 (60.0)	15 (60.0)	7 (50.0)	14 (66.7)
Private	15 (25.0)	6 (24.0)	3 (21.4)	6 (28.6)
Both Public & Private	9 (15.0)	4 (16.0)	4 (28.6)	1 (4.8)
**Years in current position**				
5 or less	4 (6.7)	3 (12.0)	1 (7.1)	0 (0.0)
5–10	8 (13.3)	4 (16.0)	2 (14.3)	2 (9.5)
> 10	48 (80.0)	18 (72.0)	11 (78.6)	19 (90.5)
**Copper IUD insertions in last 6 months**				
None	9 (15.3)	4 (16.0)	3 (23.1)	2 (9.5)
1–10	20 (33.9)	10 (40.0)	3 (23.1)	7 (33.3)
11–50	22 (37.3)	7 (28.0)	4 (30.8)	11 (52.4)
> 50	8 (13.6)	4 (16.0)	3 (23.1)	1 (4.8)
**Previous digital training experience**	15 (25.0)	3 (12.0)	4 (28.6)	8 (38.1)

### Knowledge scores

**Fig. 3:**
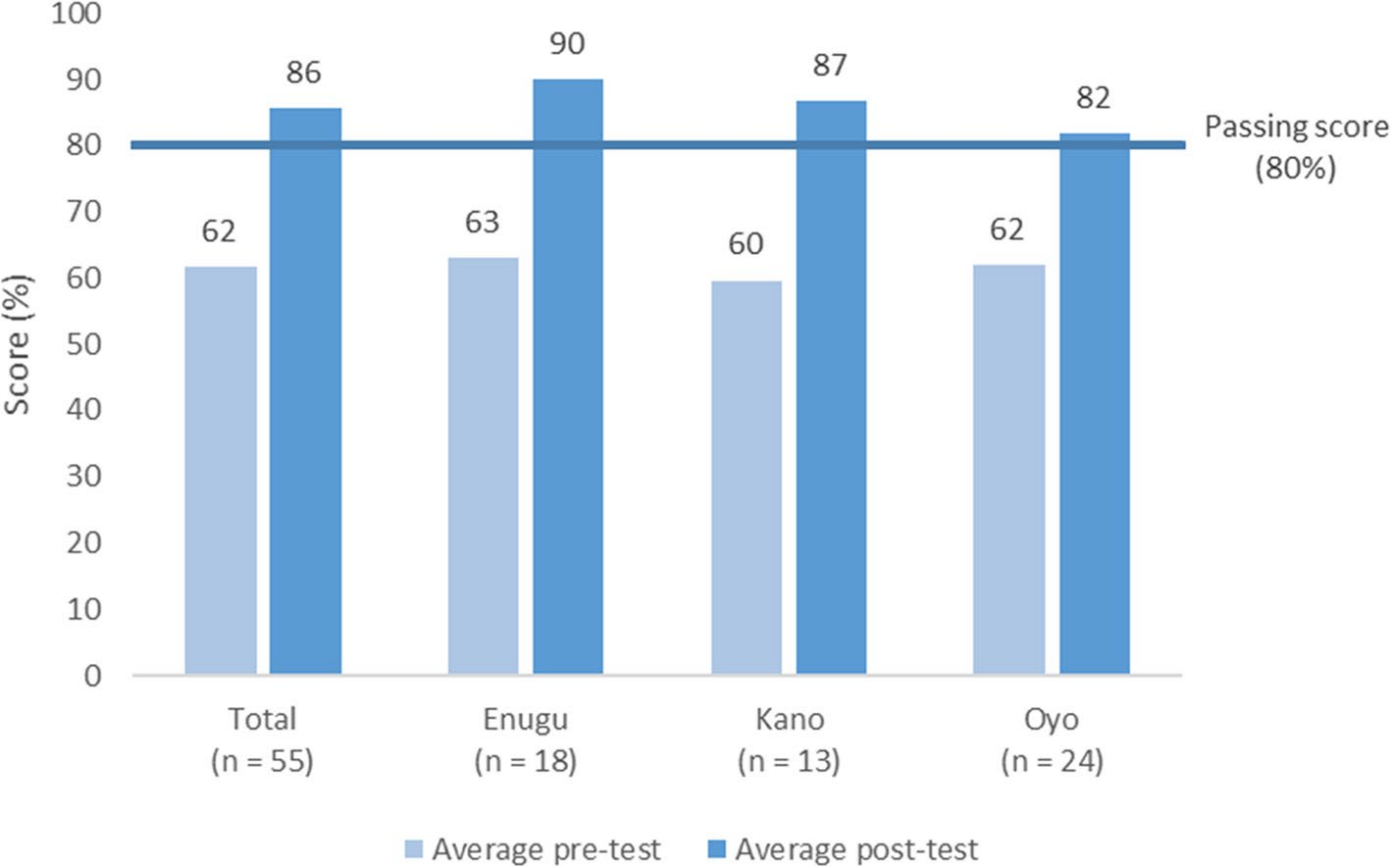
Average Pre- and Post-Training Knowledge Assessment Scores by State among family planning providers who were trained on hormonal IUD insertion using a hybrid digital approach in Nigeria. Across all states, the average pre-training knowledge assessment score was 62% and the average post-training score was 86%. None of the providers achieved the passing score of 80% or higher in the pre-training knowledge assessment, while most achieved a passing score on the post-training assessment

The average pre-training knowledge assessment score was 62% (*n* = 55, range: 39–76%) and the average post-training score was 86% (*n* = 55; range: 53–100%) (Fig. 3). None of the providers achieved the passing score of 80% or higher in the pre-training knowledge assessment, while 40 (73%) achieved a passing score on the post-training assessment. On average, providers scored 24 percentage points higher on the post-test, relative to pre-test (range: increase of 9–44 points).

### Competency following training

All 62 providers consented to their OSCE data being used for research purposes. OSCE scores (study proxy for competency) were high among all trainees. The average OSCE score across all three categories (counseling, insertion, and removal) was 95% (*n* = 62; range: 82–100%) (Table 2). Scores within categories were similarly high: counseling: 95% (range: 81–100%), insertion: 95% (76–100%), and removal: 94% (82–100%). Two people failed the OSCE on their first attempt, due to missing a required step (not overall low scores). If trainees failed in one of the OSCE stations, they were allowed to attempt the OSCE a second time. All trainees passed upon the second attempt.

**Table 2:** Average OSCE Scores by State of family planning providers who were trained on hormonal IUD insertion using a hybrid digital approach in Nigeria

	**Total**	**Enugu**	**Kano**	**Oyo**
	**(*n* = 62)**	**(*n* = 23)**	**(*n* = 14)**	**(*n* = 25)**
	Average (range), %	Average (range), %	Average (range), %	Average (range), %
**Counseling station**	95 (81–100)	90 (81–100)	98 (94–100)	98 (94–100)
**Insertion station**	95 (76–100)	90 (76–100)	98 (92–100)	98 (92–100)
**Removal station**	94 (82–100)	89 (82–100)	97 (91–100)	97 (86–100)
**All three stations**	95 (82–100)	90 (82–100)	90 (95–100)	90 (91–100)

Average OSCE scores across three categories (counseling, insertion, and removal) by state (Enugu, Kano, Oyo). The average OSCE scores was high across categories (95%) and within categories: counseling: 95% (range: 81–100%), insertion: 95% (76–100%), and removal: 94% (82–100%)

### Feasibility

Most provider trainees took the online course using a smartphone (96%), with very few reporting using a laptop (4%) or tablet (2%) to complete the training (Table 3). Most reported taking the course after working hours (76%) and at home (81%). Almost all provider trainees (95%) reported experiencing at least one technical challenge while taking the digital training, primarily related to internet connection problems (75%), bandwidth issues or slow connections (32%). Providers also reported log-in problems (61%) and difficulty navigating the platform (29%).

**Table 3:** Provider trainee views of the digital course from exit interview among participants who were trained on hormonal IUD insertion using a hybrid digital approach in Nigeria

	**Total**	**Enugu**	**Kano**	**Oyo**
	***n* = 56**	***n* = 23**	***n* = 12**	***n* = 21**
	N (%)	N (%)	N (%)	N (%)
**Device used for training**^a^				
Smartphone	54 (96.4)	23 (100.0)	11 (91.7)	20 (95.2)
Laptop	2 (3.6)	1 (4.3)	0 (0.0)	1 (4.8)
Tablet	1 (1.8)	0 (0.0)	1 (8.3)	0 (0.0)
Other	1 (1.8)	0 (0.0)	0 (0.0)	1 (4.8)
**Main time for training**				
Before working hours	3 (5.4)	2 (8.7)	0 (0.0)	1 (4.8)
During working hours	5 (8.9)	1 (4.3)	1 (8.3)	3 (14.3)
After working hours	39 (69.6)	15 (65.2)	10 (83.3)	14 (66.7)
On the weekends	5 (8.9)	2 (8.7)	1 (8.3)	2 (9.5)
No response	4 (7.1)	3 (13.0)	0 (0.0)	1 (4.8)
**Main location for training**				
At home	45 (80.4)	2 (8.7)	0 (0.0)	1 (4.8)
At work	9 (16.1)	1 (4.3)	1 (8.3)	3 (14.3)
Other	1 (1.8)	15 (65.2)	10 (83.3)	14 (66.7)
No response	1 (1.8)	2 (8.7)	1 (8.3)	2 (9.5)
**Digital training is as good as in-person training**				
Strongly disagree	4 (7.1)	2 (9.5)	1 (8.3)	1 (4.8)
Somewhat disagree	5 (8.9)	2 (9.5)	0 (0.0)	3 (14.3)
Neutral	4 (7.1)	2 (9.5)	0 (0.0)	1 (4.8)
Somewhat agree	17 (30.4)	6 (28.6)	5 (41.7)	5 (23.8)
Strongly agree	25 (44.6)	9 (42.9)	6 (50.0)	10 (47.6)
No response	1 (1.8)	0 (0.0)	0 (0.0)	1 (4.8)
**Most liked aspect of training**				
Convenient location	16 (28.6)	6 (26.1)	4 (33.3)	6 (28.6)
Less risk of COVID-19 exposure	13 (23.2)	6 (26.1)	2 (16.7)	5 (23.8)
Self-guided/self-paced	11 (19.6)	6 (26.1)	3 (25.0)	2 (9.5)
Could reference sections again	5 (8.9)	0 (0.0)	1 (8.3)	4 (19.0)
Flexible schedule	4 (7.1)	1 (4.3)	1 (8.3)	2 (9.5)
Other	6 (10.7)	3 (13.0)	1 (8.3)	2 (9.5)
No response	1 (1.8)	1 (4.3)	0 (0.0)	0 (0.0)
**Least liked aspect of training**				
No immediate feedback/immediate answers for questions	12 (21.4)	6 (26.1)	2 (16.7)	4 (19.0)
Platform difficult to navigate	11 (19.6)	6 (26.1)	1 (8.3)	4 (19.0)
Need for internet connection	9 (16.1)	3 (13.0)	1 (8.3)	5 (23.8)
Fewer professional networking opportunities	8 (14.3)	3 (13.0)	4 (33.3)	1 (4.8)
Lack of printed materials	6 (10.7)	1 (4.3)	0 (0.0)	5 (23.8)
Other	4 (7.1)	0 (0.0)	4 (33.3)	0 (0.0)
None	6 (10.7)	4 (17.4)	0 (0.0)	2 (9.5)
**Technical challenges faced during training**^a^				
Connection problems	40 (71.4)	14 (60.9)	9 (75.0)	17 (81.0)
Log-in problems	33 (58.9)	16 (69.6)	5 (41.7)	12 (57.1)
Bandwidth issues/slow connection	18 (32.1)	8 (34.8)	1 (8.3)	9 (42.9)
Unfamiliar with technology/trouble navigating the platform	16 (28.6)	3 (13.0)	3 (25.0)	10 (47.6)
Access to internet-enabled device	14 (25.0)	4 (17.4)	3 (25.0)	7 (33.3)
Training would not load	11 (19.6)	6 (26.1)	0 (0.0)	5 (23.8)
Training crashed	5 (8.9)	3 (13.0)	0 (0.0)	2 (9.5)
None of these	3 (5.4)	1 (4.3)	1 (8.3)	1 (4.8)

^a^Multiple selections allowed

A subset of the providers (*n* = 12), all the clinical supervisors who had supervised the practicum (*n* = 6), and key stakeholders (*n* = 10) completed IDIs related to feasibility, acceptability, and scalability of the digital training. Over half of the trainee provider IDI respondents (7/12) reported network issues as one of their least liked aspects of the digital training. One provider commented,

“*In short, I liked almost everything about [the digital training]. It was only network problems that at times [would] disturb us from enjoying it more." (Public sector nurse, Enugu)*

Despite these challenges, most providers agreed that the hybrid training approach gave them sufficient opportunities to ask questions, that they got the same understanding from a digital training as an in-person training), and that they had no difficulties navigating the training platform. One provider stated:

“*We got a lot of support from our supervisors, and we were given enough time to finish the modules. We were giving 2 weeks to finish 13 modules, so even if you were to be doing one module per day, you would finish it before the deadline.… As for the supervisors, they were there. You could call any of them at any time and they would attend to you and answer all your questions immediately.” (Public sector midwife, Kano)*

### Acceptability

#### Quantitative exit interview

Of those completing the exit questionnaire (Table 3), 75% ‘agreed’ or ‘strongly agreed’ that the digital training was “just as good as in-person training.” Providers’ most-liked aspect of the training was being able to take the training at home/place of their choosing (32%), the self-guided/self-paced timing (19%) and the reduced risk of COVID-19 exposure (19%). Providers’ least-liked aspects of the digital training included challenges navigating the training platform (20%), inability to network with other providers (16%) and network/bandwidth issues (16%).

#### Qualitative IDIs

In addition to the convenience and self-guided nature of the digital training, providers in the IDIs cited the WhatsApp discussion group as a valuable component of the hybrid training. One provider described the benefits of the discussion group, and the group moderator (who were study team members and medical doctors) in particular, by saying:

“*Anytime there’s need for clarification, when we raise it on the platform—the WhatsApp group—they explain, they clarify it. They even make calls. They are readily available for us at any time…to clarify issues for us.*” *(Public and private sector midwife, Oyo)*

Several respondents not only liked the digital training, but would prefer it over traditional, classroom-based alternatives. The preference for digital over in-person training was explained by one provider trainee:

*“What I enjoyed most was that I was able to have my training at my leisure. You know, any time you are free—1 hour, 2 hours—you just log in and start doing something. There is nothing disturbing you—you have your time for it.” (Provider trainee, public and private sector employment, Kano)*

All key informants interviewed expressed support for continuing or expanding the digital training model. Reasons included cost savings, potential to expand access to FP services, and opportunity to expand method choice in Nigeria (Supplemental Materials 2). One MOH official noted:

*“So, we can…start to prepare for scale-up because we are really excited about the digital training, from the government perspective. As a policy maker, too, I am keenly interested in it. It will allow me a very rapid traction and invariably will also support access to family planning information [and] services, as well as enhancing uptake.”*

IDI participants had recommendations related to data allowances, client follow-up, and incentives. Provider trainees and clinical supervisors mentioned the need to increase the data allowance for completing the digital training, since some provider trainees took longer due to network connectivity issues.

Trainer respondents proposed decreasing the time interval between the didactic training and the live-client practicum, to mitigate issues with knowledge retention and trainee performance. Lastly, some key informants recommended providing continuing medical education (CME) credits or certificates to trainees to incentivize participation and further legitimize the training. One key informant commented:

*“If you look at the human nature, incentives do usually give… motivation to the participants… so it’s a good idea. Honestly, it will motivate the participants. CME [is a good idea], especially for the professional bodies that use that. You see, for doctors, anywhere there is CME, we run there. You need CME for your annual registration.” (Key informant, Kano state MOH)*

According to some of the providers and key informants, the lack of per diems for digital or hybrid training could make the need for non-monetary incentives, such as certificates or data bundles, quite important.

“*[Incentives play a] very big role, na! There is nobody [who] doesn’t want an incentive...Just as I was telling you, incentive could be in any form, [not just] monetary incentives. There is nobody that will not want to be appreciated so if these providers know that they will get something, they will be more committed. …But when they know that nothing is attached some of them can even at a time abandon [the training] on the way and say, ‘I am tired of this thing’.”* (*Key informant, Enugu)*

### Costs

**Fig. 4:**
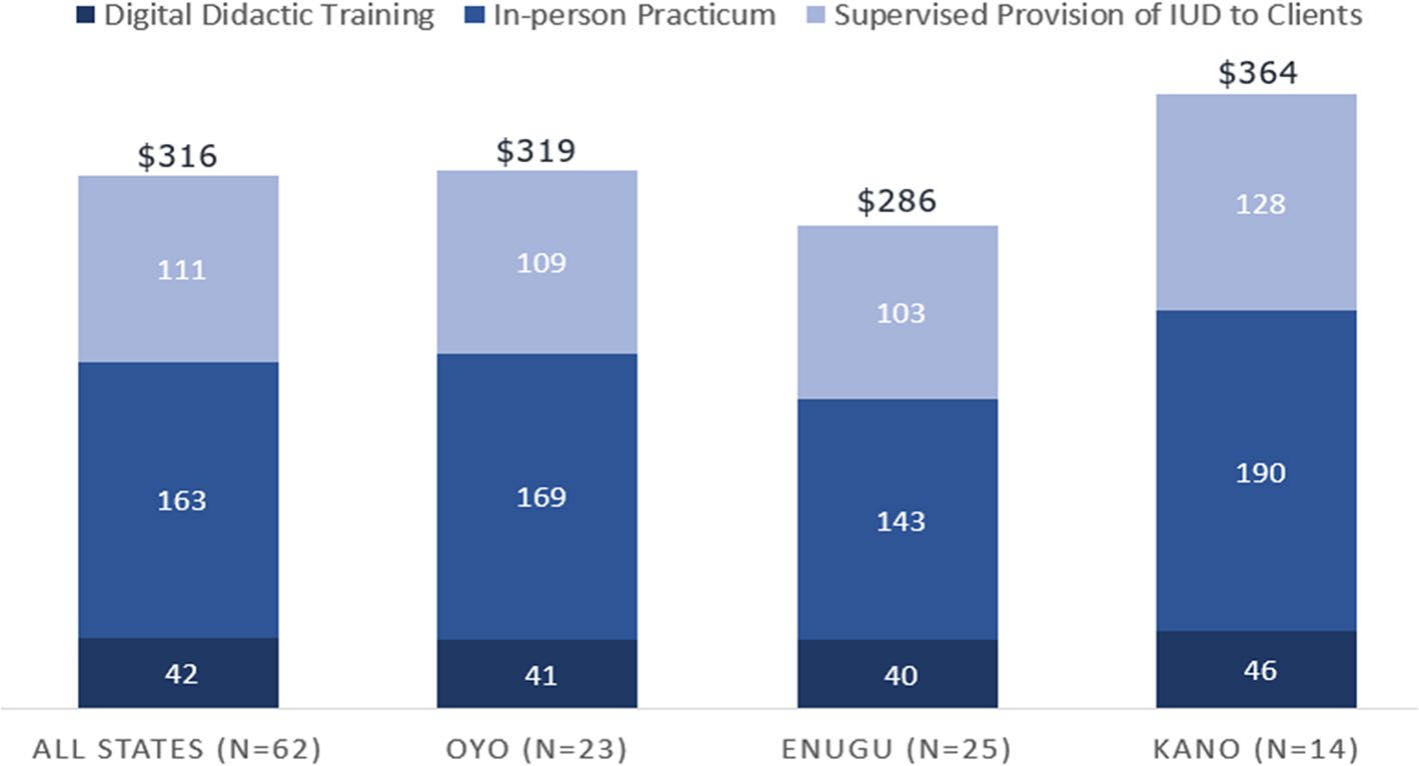
Cost of Hormonal IUD Hybrid Digital Training in Enugu, Oyo, and Kano States, in US Dollars Across all states, the cost per provider trained for the hormonal IUD hybrid digital training was $316. By state, the training costs varied with Enugu reporting the lowest cost per provider ($286), followed by Oyo ($319) and Kano ($364). Digital didactic training component costs were similar across states and range between $40–46

Overall, the cost per provider trained for the hormonal IUD hybrid digital training was $316 (131,003 NGN) across all study settings (*n* = 62 providers trained) (Fig. 4). The training costs varied slightly by state, with Enugu (*n* = 25) reporting the lowest per provider cost of $286 (118,667 NGN) compared with $319 (132,351 NGN) in Oyo (*n* = 23) and $364 (150,818 NGN) in Kano (*n* = 14) Costs associated with the digital didactic training component were similar across states (range between $40–46). There was greater variability in the in-person practicum costs (range from $143-$190) and the live client training costs (range from $103–128). In these cases, staff time and materials were generally consistent across sites, with most of the variability attributed to differences in the cost of meeting venues and travel within different states.

## Discussion

This mixed methods study examined the feasibility, acceptability and cost-effectiveness of hybrid digital training approach on hormonal IUD in Nigeria for family planning providers. The study found that significant knowledge gains occurred following the training, from an average of 62% at baseline to 86% at endline. Qualitative and quantitative findings also indicate high levels of acceptability of the hybrid approach from both providers and stakeholders, who also felt this training approach was feasible, scalable, and of high quality. Our costing data also suggest that the hybrid approach is likely cost-saving relative to a fully in-person training alternative.

Digital training approaches for health care providers are recommended by the HIP Partnership as a High Impact Practice enhancement, to complement in-person health care provider training [14]. Recent systematic reviews from LMICs found that medical and nursing students found that digital training tools are acceptable, particularly for efficiency and personalizing content [15]; that digital learning was as effective as in-person learning approaches for increasing knowledge [16]; and was associated with increases in health worker motivation, self-efficacy, and job satisfaction [17]. Digital training has been used to educate providers in Liberia on Ebola virus [18], to provide in-service training using online videos on child health services on mobile phones in Nigeria [19], and to provide FP refresher training on management of contraceptive side effects and misconceptions using interactive voice response and SMS in Senegal [20]. And in China, a mobile phone-based video training improved provider’s knowledge and attitudes towards LARCs and increased coverage of LARCs among women attending post-abortion care services [21]. Hybrid digital/in-person training approaches are particularly aligned with in-service training on hormonal IUDs, given the overlay of the COVID-19 pandemic with hormonal IUD scale up, and the need for both didactic training and in-person supervised service provision [22].

Scaling effective and efficient digital training interventions will be dependent on addressing barriers, including technical challenges. Confirming previous studies [23], our study found technical challenges (including low bandwidth, limited network coverage, and slow data speeds) to be substantial barriers to training completion. Three quarters of the providers in our study were inexperienced with digital trainings. Our study supported provider trainees by providing a WhatsApp forum and a synchronous session to answer questions. Design of the platform is also important. Previous studies recommend development be driven by three key priorities: simplicity, interoperability, and adaptability [24]. Simpler systems may be easier to scale, and interoperable, open-source platforms may provide efficiencies that reduce costs and ultimately enable greater impact of the training.

As digital FP training interventions begin to be more frequently used, more rigorous research is needed on their effect/impact on contraceptive method availability, provider satisfaction, and quality of patient care [25]. Additional research topics of interest may include the role of digital or hybrid approaches in supportive supervision and clinical supervisor training. Heterogeneity across cadres and sectors, knowledge retention, the use of digital platforms for periodic refresher trainings are also highly relevant areas for investigation. Ministries of Health may want to integrate use of digital training into existing in-service training databases or tracking systems.

Limited evidence is available related to costs of digital health interventions for LARCs [26, 27], and for digital trainings more specifically. However, digital or hybrid digital/in-person health trainings may potentially be cost-saving as compared to face-to-face training alternatives. In this study, we found that the cost to train a provider using the hybrid digital training approach was $316. This is considerably lower than the cost ($426) per provider trained in an in-person hormonal IUD training conducted in Nigeria in 2017 (data re-analyzed from Brunie et al., 2020). The training conducted in 2017 utilized a similar model (didactic training, in-person practicum, and supervised provision of services to clients) [8] and differed in that the didactic training was done in-person rather than using a digital platform. Additional research will be useful to examine the relative cost-effectiveness of digital or hybrid digital/in-person training approaches relative to in-person alternatives, including understanding differences among different cadres of providers as well as providers who are previously LARC-trained in comparison to those with no LARC training.

There is great potential, and great will, to use digital and hybrid digital/in-person training approaches to improve access to FP in Nigeria. In the FP2030 Commitment of Nigeria, digital training is named as an approach to improve access and choice of FP adoption [28]. Internet penetration in Nigeria was at 51%, with over 100 million users in 2022 [29]. Over 84% of internet traffic is currently generated by mobile devices [29]. This synergy of key stakeholder support and high phone saturation has had noticeable effects: since completion of the study, the federal MOH has decided to incorporate the hybrid digital training approach into national hormonal IUD scale-up in Nigeria (personal communication, Dr. Afolabi, 2022) and use of the digital training platform is being explored in other contexts [30].

### Limitations

This study was relatively small, enrolled health care providers who were purposively selected, and included only three states in Nigeria, limiting generalizability to the study sample. However, the scale of the study matches other studies in the literature around hybrid digital training approaches, which generally present small pilot projects with limited exploration of intervention effectiveness [27]. The health care providers trained were experienced in provision of non-hormonal IUD, and digital training approaches may be significantly different for providers not experienced in or trained on non-hormonal IUD. Additionally, our ability to draw conclusions about potential cost savings is limited by not having a comparison group, and our considering only direct training costs. Despite these limitations, we feel that the study provides initial evidence for the potential success in application of the digital training approach for hormonal IUD in Nigeria.

## Conclusion

As countries expand their FP method mix to provide a broader range of contraceptive options, identifying feasible, acceptable, and cost-efficient in-service training approaches for FP providers will be a cornerstone of high-quality service provision. The findings from this study provide support the potential use of a hybrid digital and in-person training approach for hormonal IUD for LARC-experienced health care providers. We did not find any concerns in the use of this approach and believe it can be useful in other settings. As the federal MOH and other key stakeholders in Nigeria incorporate digital training into hormonal IUD scale up, future research is still needed on the effectiveness of the training approach at scale, on skills retention, on reduction of barriers to use by health care providers, and, importantly, on the use of hybrid training models among LARC-inexperienced providers.

### Supplementary Information

**Additional file 1.** **Supplemental Materials 1. **The hybrid digital training model consists of a digital didactic training (stage 1), practice on models through in-person practicum (stage 2), and practice on live clients (stage 3).

**Additional file 2.** **Supplemental Materials 2. **Key quotes related to themes of feasibility, scalability, and opportunities to improve digital health trainings from the prospective of providers, trainers, and other key informants in Nigeria.

## Data Availability

The de-identified quantitative datasets generated from the study, the quantitative/qualitative survey codebooks, consent forms, and tools are publicly available on Harvard Dataverse: https://doi.org/10.7910/DVN/4PHEUT . The qualitative datasets are not publicly available since they could not be fully de-identified.

## Footnotes

1 LARC service provision, including IUDs, is currently limited to doctors, nurses, midwives, and Senior Community Health Extension Workers (SCHEWs) in Nigeria.
