# Supplementary material for: Use of a hybrid digital training approach for hormonal IUD providers in Nigeria: results from a mixed method study
Source: BMC Health Serv Res. 2023 Nov 29;23:1316. doi: 10.1186/s12913-023-10211-5 (PMC10685471; doi:10.1186/s12913-023-10211-5)

**Supplemental Materials 1 title:** Hybrid Digital Training Description

**Supplemental Materials 1 caption:** the hybrid digital training model consists of a digital didactic training (stage 1), practice on models through in-person practicum (stage 2), and practice on live clients (stage 3).


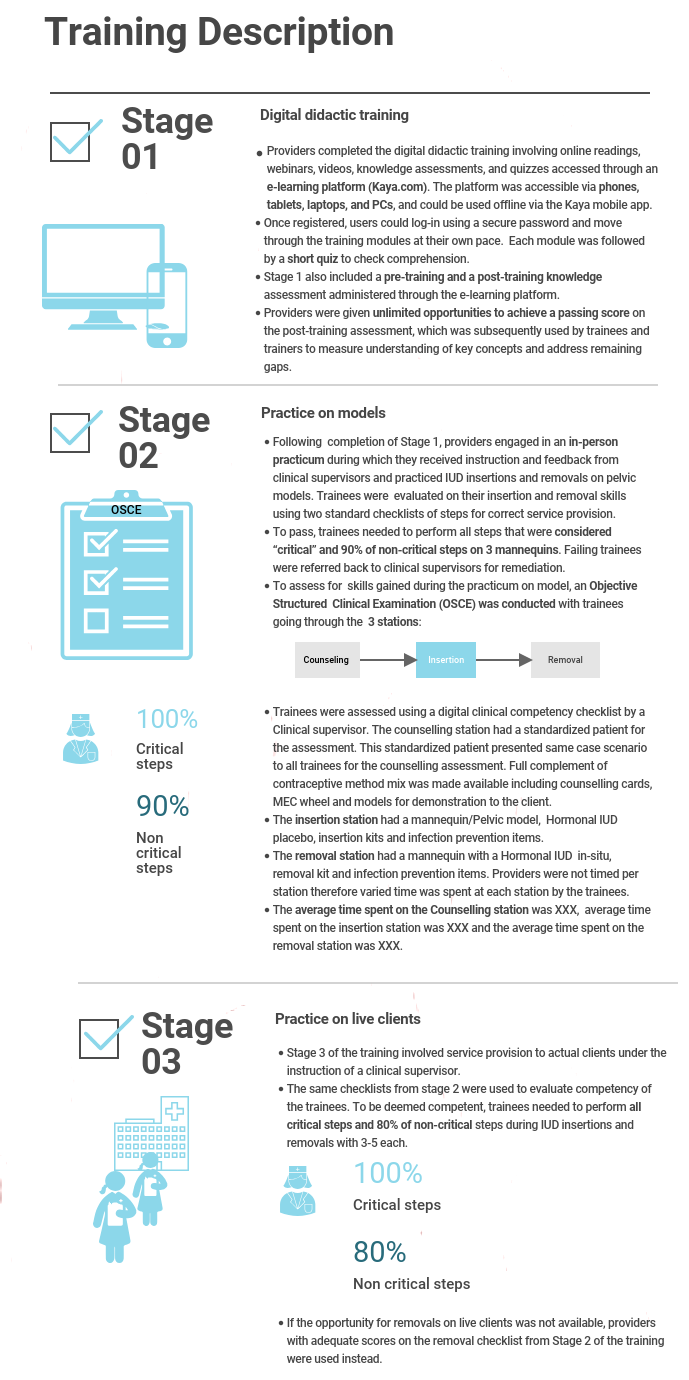

Supplement: Supplementary file 1 — Additional file 1. Supplemental Materials 1. The hybrid digital training model consists of a digital didactic training (stage 1), practice on models through in-person practicum (stage 2), and practice on live clients (stage 3). [file 12913_2023_10211_MOESM1_ESM.docx]
