# Supplementary material for: Use of a hybrid digital training approach for hormonal IUD providers in Nigeria: results from a mixed method study
Source: BMC Health Serv Res. 2023 Nov 29;23:1316. doi: 10.1186/s12913-023-10211-5 (PMC10685471; doi:10.1186/s12913-023-10211-5)
Supplement: Supplementary file 2 — Additional file 2. Supplemental Materials 2. Key quotes related to themes of feasibility, scalability, and opportunities to improve digital health trainings from the prospective of providers, trainers, and other key informants in Nigeria. [file 12913_2023_10211_MOESM2_ESM.docx]

**Supplemental Materials 2 title:** Qualitative Findings on Feasibility, Scalability, and Opportunities to Improve Digital Health Trainings

**Supplemental Materials 2 caption:** Key quotes related to themes of feasibility, scalability, and opportunities to improve digital health trainings from the prospective of providers, trainers, and other key informants in Nigeria.

| **Theme** | **Illustrative Quote** | **Source** |
| --- | --- | --- |
| **Feasibility** | *“Everything was easy for me … the video, the audio and the written....the Zoom connects more people together, we are able to have different challenges from different people which were being attended to by the coordinators.”*  *“I think the digital trainings, they have a lot of advantages; number 1 is that you spend less, and the trainings are not all that long, though it is done at your own pace, nobody forces you, give you time, you do it at your own pace. So it’s an advantage because you do it when you are free. It’s a very good advantage. And what else again, so the learnings, if you really want to learn, you learn because what you don’t understand, you go back and replay it and you still get whatever you want, so I think it’s an advantage.”*  *“In my own opinion, the digital training is a welcomed development especially now that we have pandemic like COVID-19 that has brought restriction to gatherings... you can train so many people at a time without bringing them together ... and another thing is the digital training gives the healthcare worker or the trainee like ample time rather than if you compare it with the physical training. For instance, in the physical training, because we don’t really want to take the healthcare worker off their duty station, we usually compress the training into like 4-5 days at most a week, but for the digital training they usually have like more time, like this one, it was like 2 weeks, we were given like 2 weeks which is enough for them to go through the training... And I think the understanding too with comprehension will be better. Also, with regards to their like place of work, you don’t take them off their duty station. For the digital training, it is something that they can do at their own free time you understand... It does not affect their work. So, I think these are the main advantages it has over the physical training.”* | Provider  Key Informant    Key Informant |
|  |  |  |
| **Acceptability** | *“The e-learning training is quite more comfortable for us because of our working condition and leaving our family to go to other places to stay two or three days coming back, instead, you will be at your home, doing everything at your leisure time, even after, even when you finish every work you want to do, but with the e-learning, you can continue even till late at night to do your work. So I prefer e-learning to that classroom work"* | Public and Private Sector Provider (Enugu) |
|  | *“In the digital you know is that you have everything you can still go back. Even if they are practicing in the hospital and need to clarify something they can still go back to the video and look at it. "* | Clinical Supervisor |
|  | *“It was excellent. They were real prepared based on the information about what to do...And during the practical experience with the participant they were ready to learn because on the day of their practical they came early despite that they came from home they were ready to learn even they were not ready to go for tea break they were there to practice until they got it. They were very happy"* | Clinical Supervisor |
|  | *“I like everything about it because I choose the time that is conducive for me and [the training staff] are very kind-they answered all our questions”.* | Public sector provider (Enugu) |
| **Scalability** | *“You know before the digital training [pilot], we had a lot of fears [about it], but when we were at the platform, all those fears just wiped off. So, Kaya or any other digital platform, if it is possible, information will be passed to this one, people will get more knowledge instead of waiting for more funding, and funding, even when funding is not coming forth. I think it is cost effective and we can still go by it.”* | Key informant |
| **Opportunities for Improvement** | *“Yes, there should be availability of Network. They should use one network that is available so that anybody can connect to and secondly the [data stipend] should be increased”* | Public Sector Provider (Kano) |
|  | *“My suggestion is to make sure that if there is any opportunity for data stipend, at least to get to all the people that will be involved in the digital training so that they will not have any excuse to give, and that before selecting participants for digital training the family planning coordinator should make sure that she selects facilities that have workable android phones because we noticed that some android phones were not able to carry the weight of the training and some people were just on and off, on and off, running about to see where they can get… but if you have a good android phone, wherever you are, it will just catch up with the network and the whole thing will flow very well.”* | *Clinical Supervisor* |
|  | *“The suggestion I have is that supervision on live client should start immediately after the practicum, so they won’t forget what they have learnt.”* | *Clinical Supervisor* |
